# Change processes during intensive day programme treatment for adolescent anorexia nervosa: a dyadic interview analysis of adolescent and parent views

**DOI:** 10.3389/fpsyg.2023.1226605

**Published:** 2023-08-10

**Authors:** Amy Colla, Julian Baudinet, Penny Cavenagh, Hugo Senra, Elizabeth Goddard

**Affiliations:** ^1^School of Health and Social Care, University of Essex, Colchester, United Kingdom; ^2^Essex Partnership University NHS Trust, Runwell, United Kingdom; ^3^Maudsley Centre for Child and Adolescent Eating Disorders (MCCAED), Maudsley Hospital, London, United Kingdom; ^4^Institute of Psychiatry, Psychology and Neuroscience (IoPPN), King's College London, London, United Kingdom; ^5^Suffolk Doctoral College, University of Suffolk, Ipswich, United Kingdom; ^6^CICECO Aveiro Institute of Materials, University of Aveiro, Aveiro, Portugal

**Keywords:** anorexia nervosa, day programme, intensive outpatient treatment, qualitative analysis, dyadic interview analysis, change processes, eating disorder treatment, adolescents

## Abstract

**Background:**

Day programmes for adolescent anorexia nervosa (AN) can function as an alternative to inpatient admissions and/or an increase in outpatient treatment intensity. Processes of change during treatment for AN are currently poorly understood. This study aimed to explore how adolescents with AN and their parents understood the helpful and unhelpful factors and processes that impacted them during day programme treatment.

**Method:**

A critical realist paradigm was used to qualitatively explore the views of 16 participants. Participants were recruited from the Intensive Treatment Programme (ITP) at the Maudsley Center for Child and Adolescent Eating Disorders (MCCAED) at the end of treatment. Dyadic Interview Analysis (DIA) was used to compare and contrast the narratives of the seven adolescent–parent pairs after two inductive reflexive thematic analyses were conducted for the group of eight adolescents and the group of eight parents separately.

**Results:**

Eight subthemes across three themes were identified: 1) “*Like me she didn't feel so alone anymore*”—families connect with staff, peers, and each other; 2) “*You have to eat because ITP say so*”—the programme provides families with containment through its structure and authority; and 3) “*I found that I was using the skills I learnt there like in multiple aspects of my life, not just around food”*—families take in new ideas and generalize these into their lives. These interconnected themes generated hope and change. However, helpful elements individually could be unhelpful if one or more of the other factors were missing. For example, staff firmness, which participants often found helpful (theme two), could be experienced as harshness when adolescents did not feel related to as individuals (theme one).

**Conclusion:**

The findings can be conceptualized within recent descriptions regarding the therapeutic change, including epistemic trust and mentalization. Treatment characteristics, such as intensity and containment, as well as illness-specific factors and processes, such as control and collaboration, the role of peer support, and the potential for family members to experience the impact of the adolescent's AN and treatment non-response as traumatic, are equally important to consider.

## Background

Day programmes are becoming increasingly widespread internationally in the treatment of adolescent eating disorders (Baudinet and Simic, 2021). They offer relatively brief, intensive treatment for those who require more support than can be offered in the typical outpatient treatment context. They go by several different names, including day programmes, day treatments, intensive outpatient programmes, and partial hospitalization programmes, and vary considerably between centers. Differences include the theoretical model of treatment (e.g., predominantly influenced by family therapy models, cognitive behavior therapy, etc.), age of service users (adolescent only, all ages, adult only, etc.), diagnostic mix (single diagnosis vs. mixed diagnosis), and duration of treatment.

Despite these differences, all day programmes share two common features: (1) all offer more intensive support and clinical contact compared to outpatient treatment and (2) service users do not stay overnight in the facility, as per inpatient or residential treatment. Most function as both a step-up in treatment intensity, if more is needed than can be offered in the outpatient setting, as well as a step-down from more intensive treatments (e.g., inpatient admissions) to aid the often difficult transition back into the community.

In recent years, there have been many studies investigating adolescent eating disorder day programme treatment outcomes, typically in the form of uncontrolled case series. They are associated with physical health improvements and weight gain (for those needing to), reduced eating disorder psychopathology, improvements in co-morbid anxiety and depression, and shifts in family functioning. These outcomes are generally maintained at short- and long-term follow-ups, and outcomes are similar to inpatient treatment after brief stabilization (Herpertz-Dahlmann et al., 2014; Baudinet and Simic, 2021).

Despite this emerging empirical evidence base, little attention has been given to the qualitative experience of adolescents and families in day programme treatment for AN. Parks and colleagues (Parks et al., 2016) explored adolescents' (*N* = 29, age 11–18) impressions of involving family members in their mixed-diagnosis day programme treatment using Likert scale and open-ended questionnaire data. They found that many were reluctant to involve family members at the beginning of treatment, but by the end of treatment, most said they appreciated it and that their family relationships had improved. Findings suggest that involving parents in day programme treatment is useful; however, little more is known beyond this about the experience of treatment or how adolescents and families perceive treatment to promote change.

This study aimed to explore how adolescents with anorexia nervosa and their parents understood the helpful and unhelpful aspects and processes that impacted them during day programme treatment.

## Method

This study was approved by the London-Hampstead Ethics Committee (REC: 19/LO/0766). Written informed consent was provided by all participants in this study. For those under 16 years of age, parental consent was also provided in addition to adolescent assent.

### Design

Information about the perceived change processes during an intensive day treatment programme (ITP) at the Maudsley Center for Child and Adolescent Eating Disorders (MCCAED), UK, was gathered using individual qualitative interviews (~60 m), conducted face-to-face (in the clinic) or via video-call by the lead author, AC. The mode of the interview was based on participant preference. All were semi-structured and followed the same topic guide (see Supplementary material), which included general questions such as their experience of treatment and the changes they observed occurring during treatment (and their theories regarding these changes). There were also prompts if a theoretically relevant concept was not spontaneously raised by participants, including intensity of programme, being part of a group, family relationships, understandings of AN, boundaries and rules, and specific programme elements. After the first four paired interviews, motivation and hope were added as additional prompts. All interviews were audio-recorded and transcribed verbatim.

Researchers gathered the following information about the adolescents from their clinical records: age, ethnicity, length of treatment, previous treatments, and the service's outcome classification based on the Morgan–Russell Outcome Assessment Schedule (Morgan and Hayward, 1988). The descriptors (Poor, Intermediate, and Good; see Table 1 for further information) are based on physical and behavioral criteria and do not consider psychological aspects of recovery; therefore, they did not necessarily correspond to participants' subjective perceptions of recovery (gathered via the interviews).

**Table 1:** Participant demographics.

**Participant pseudonym(s) (adolescent and parent)**	**Age^a^**	**Length of treatment (weeks)^b^**	**Morgan–Russell outcome^c^**	**Previous treatments^d^**
“Alice” and “April”	17	20	Poor	Inpatient
				Outpatient ED
				MFT-AN (during ITP)
“Bella” and “Bridget”	17	13	Good	Pediatric
				Outpatient ED
“Clara^e^” and “Carol”	16	25	Poor	Outpatient ED
				Pediatric MFT-AN (during ITP)
		17	Intermediate	Inpatient
“Daniela” and “Dawn”	15	16	Good	CAMHS
				Outpatient ED
				Pediatric
				MFT-AN (during ITP)
“Evie” and “Eleanor”	16	14	Good	Outpatient ED
“Fae” and “Fran”	16	5	Good	Outpatient ED
				MFT-AN
“Gemma” and “Greg”	14	14	Good	Outpatient
“Helen” (parent)	16	26	Intermediate	Outpatient ED
				Pediatric
				MFT-AN (during ITP)
“Ilana” (adolescent)	17	12	Poor	Pediatric
				Outpatient ED

a Age of the adolescent at the time of participation.

b NB, some adolescents attended full-time while others attended part-time for some or all of their treatment duration.

c Good: >85 %mBMI with menstruation or premenarchal and no bulimic symptoms; Intermediate: >85 %mBMI without menstruation or bulimic symptoms averaging <1 per week over the last month; Poor: < 85% without menstruation or bulimic symptoms averaging ≥1 per week over the last month).

d Reported by Families themselves. Options include outpatient children and adolescents eating disorder service (“outpatient ED”), inpatient general psychiatric ward or eating-disorder-specific ward (“inpatient”), Pediatric hospitalization (“Pediatric”), Multi-family Therapy (“MFT”), and generic Child and Adolescent Mental Health Services (CAMHS).

e Clara had two episodes of treatment within the programme, before and after inpatient admission.

### Sample and recruitment process

A convenience sample of adolescents in treatment at ITP and their parents was used in this study, whereby all participants who met eligibility criteria during the recruitment phase (August–September 2019) were invited to participate. Adolescents and their parents were eligible for this study if the adolescent: (1) had received a DSM-5 (American Psychiatric Association, 2013) diagnosed of anorexia nervosa, atypical anorexia nervosa, or related restrictive eating disorder; and (2) was aged 12–17 years, inclusive. Participants were initially approached by a member of the clinical team. Once consent for contact by the researchers was obtained, the clinical team was no longer involved in the process. They were approached at the end of their ITP treatment (between 2 weeks prior and 2 months after discharge).

### Day programme treatment description and service context

ITP is a day programme in the Maudsley Center for Child and Adolescent Eating Disorders (MCCAED) in South London, UK. ITP accepts referrals from across England and is principally aimed at adolescents with restrictive eating patterns whose outpatient treatment is not progressing and need a more intensive approach. Its primary aim is to offer an alternative to inpatient treatment. ITP operates 5 days per week (Monday–Friday) offering a combination of individual, family, and group therapy, meal and dietetic support, as well as education support. It is highly structured, with attendance titrating down over the course of the programme.

Adolescents attend education sessions and therapeutic group sessions, including Radically Open Dialectical Behavior Therapy adapted for adolescents [RO-DBT (Lynch, 2018; Baudinet et al., 2020)] to address emotional and behavioral over-control; two Cognitive Behavior Therapy (CBT) groups for clinically significant perfectionism and generalized anxiety; Cognitive remediation Therapy [CRT (Maiden et al., 2014)] to address cognitive inflexibility; art therapy to provide a non-verbal therapeutic space; yoga therapy for anorexia nervosa to promote interoceptive awareness and experience the body differently; a narrative therapy group called “Who do you think you are?” to explore identity outside of their eating disorder; food group to learn about nutrition and how to make balanced meals; and a group called “joining the dots” focusing on school reintegration. Each family is assigned a “mini-team” of two professionals who coordinate their care and provide individual therapy for the adolescent as well as family sessions. Family sessions are concordant with the Maudsley model of FT-AN (Eisler et al., 2016) but may require adaptation and flexibility given that the families may not have responded to outpatient FT-AN.

Further details of the programme and outcomes are published elsewhere (Simic et al., 2018; Baudinet et al., 2020). The wider MCCAED service is a large, well-established specialist child and adolescent eating disorders service that offers a range of other outpatient and intensive outpatient treatments (Simic et al., 2022; Stewart et al., 2022).

### Data analysis

Data were analyzed from a critical realist position using Dyadic Interview Analysis (DIA) (Eisikovits and Koren, 2010). DIA is a method of analysis developed for analyzing interviews involving multiple pairs of people known to each other. This involved extending reflexive thematic analysis (Braun and Clarke, 2006, 2012) to consider dyadic responding.

The first author, AC, initially conducted the first four steps of reflexive thematic analysis (Braun and Clarke, 2020) for the adolescent interviews. This involved initial familiarization with the data (step 1), generating initial codes (step 2), combining initial codes into themes and subthemes (step 3), and reviewing themes for internal and external homogeneity (step 4). These themes and subthemes were given provisional names and brief descriptions and were sent to co-authors for review and discussion.

The same 4 steps were then completed for the parent interviews. Some initial dyadic analysis occurred during these steps by having already provisionally completed the adolescent group thematic analysis. This was not considered problematic because the aim was ultimately to have a dyadic understanding of the issues.

Dyadic interview analysis was then conducted for each pair of parent and adolescent interviews. This involved (1) a review of reflexive notes made post-interview and post-transcribing, (2) a review of adolescent and parent transcripts with the generation of initial codes (adolescent only, parent only, or discussed by both), (3) sections of interviews that were coded as ‘*discussed by both'* were then divided into rough categories (e.g., before treatment, family relationships, etc.), (4) explicit agreement or disagreement between the adolescent and parent on these categories was then reviewed, and (5) a detailed narrative of each pair's narrative was written and points of mirroring and divergence highlighted.

Once those steps were completed, AC returned to the two provisional thematic analyses and considered how the understanding of the dyadic analysis of each pair might change the understanding of these themes from the two groups. AC considered whether separate themes were needed for adolescents and parents and concluded that their experiences could be captured through a single thematic structure. Steps 4 (reviewing themes for internal homogeneity and external heterogeneity) and 5 (defining and naming themes and subthemes) of reflexive thematic analysis were then completed for the combined dataset. In the end, the final themes and subthemes (akin to step 6 of reflexive thematic analysis) were presented. In practice, the process was iterative, with a return to earlier steps whenever required in consultation with co-authors.

### Reflexivity statement

AC made her position and assumptions explicit through a bracketing interview (Fischer, 2009) during the initial stages of research design, writing reflexive notes after every interview (or pairs of interviews), and completing a positionality map (Jacobson and Mustafa, 2019) before conducting the analysis. AC also consulted regularly with co-authors to check for assumptions throughout the analysis.

AC is a Clinical Psychologist (in training at the time she was completing this research) who takes a social justice and advocacy lens in her work. AC does not have a personal history of eating-related distress and her familiarity with these difficulties came primarily from having worked in the service for 2 years before training. While familiarity with the setting is desirable, AC was aware that having been part of the system might bias her interpretations. AC had worked intensively with families where the adolescent had a diagnosis of anorexia nervosa, researched different theories and approaches, and completed a meta-synthesis of qualitative research on AN, so she brought several pre-existing ideas and hypotheses about the nature of restrictive eating disorders and what enables recovery.

JB is a Clinical Psychologist who has extensive experience working with adolescents with eating disorders across inpatient, day programme, and outpatient settings. JB approaches his work with a primary focus on engagement and formulation. He is also aware of eating disorder research and theory through experience researching, teaching, and writing about eating disorders. This is part of his approach to the data and has influenced the interpretation of different individuals' comments and experiences.

AC and JB were aware that AC's prior role within the service and lack of first-hand experience with AN risked her being seen as an expert within the system by participants. It is possible that participants did not see AC in her role as an interviewer as fully independent of the service, which may have impacted their responses. AC acknowledged this with participants to facilitate more open discussions; the candid responses suggest that the approach was at least in part successful.

### Member checking

All participants agreed to be contacted once a draft of the results were completed. Those available were sent a summary of how the data were analyzed and a short version of the results. Phone calls were arranged to discuss whether the results reflected their experiences. Participants could also add further clarification to the ideas presented.

## Results

### Study participants

Twelve families attended ITP during the data collection period. One of the adolescents had a diagnosis of ARFID and was excluded. Another was in a crisis unrelated to the adolescent's eating difficulties and the clinical team deemed it inappropriate to approach them about participating.

From the 10 eligible families, a total of 16 participants (eight adolescents and eight parents), from nine families consented to participate and completed interviews. This consisted of seven adolescent–parent pairs, and one parent and one adolescent participating alone. Aliases were assigned to ensure anonymity. Each adolescent and parent from the same family were assigned a name that starts with the same letter (e.g., Alice is April's daughter, etc.). See Table 1 for demographics. All adolescents were female; there were seven mothers and one father.

Six participants expressed an interest in reviewing a summary of the draft results. Of these, one parent and one adolescent gave feedback via telephone (~45–60 min), and one parent gave feedback by email due to time constraints.

“Ilana” (A) reported that the themes captured the most important aspects of her experience of the treatment, and was surprised to find that “everyone else” felt the same as her. She stated that she had only noticed some of the negative aspects of themes at the end of treatment. “Helen” (P) strongly endorsed the idea that treatment failure was traumatic. They both identified with ideas about the difficult transition out of the programme.

### Self-reported progress

Participant narratives did not necessarily correspond to their Morgan–Russell outcome category. For example, Ilana (A), listed as having a “poor” outcome in terms of the Morgan–Russell criteria, was largely positive about her recovery and reported that she was still doing well the following year during the member-checking phase.

Participants described their situation before the programme as extremely challenging, as would be expected in the context of needing an intensive service. Narratives about the programme were overall positive, and most participants were cautiously optimistic about their future. There was some uncertainty about the solidity of their recovery, which again is to be expected on discharge from a specialist intensive service as participants were all returning to outpatient mental health services to continue treatment. Alice (A) had a distinctly more pessimistic narrative as she was aware that she had not responded to treatment to the same degree as others.

### Dyadic interview analysis findings

Three themes with eight subthemes were interpreted from the data. It was possible to generate a single set of themes from the three analyses due to the significant overlap between the narratives of the two groups of participants.

**Figure 1:**
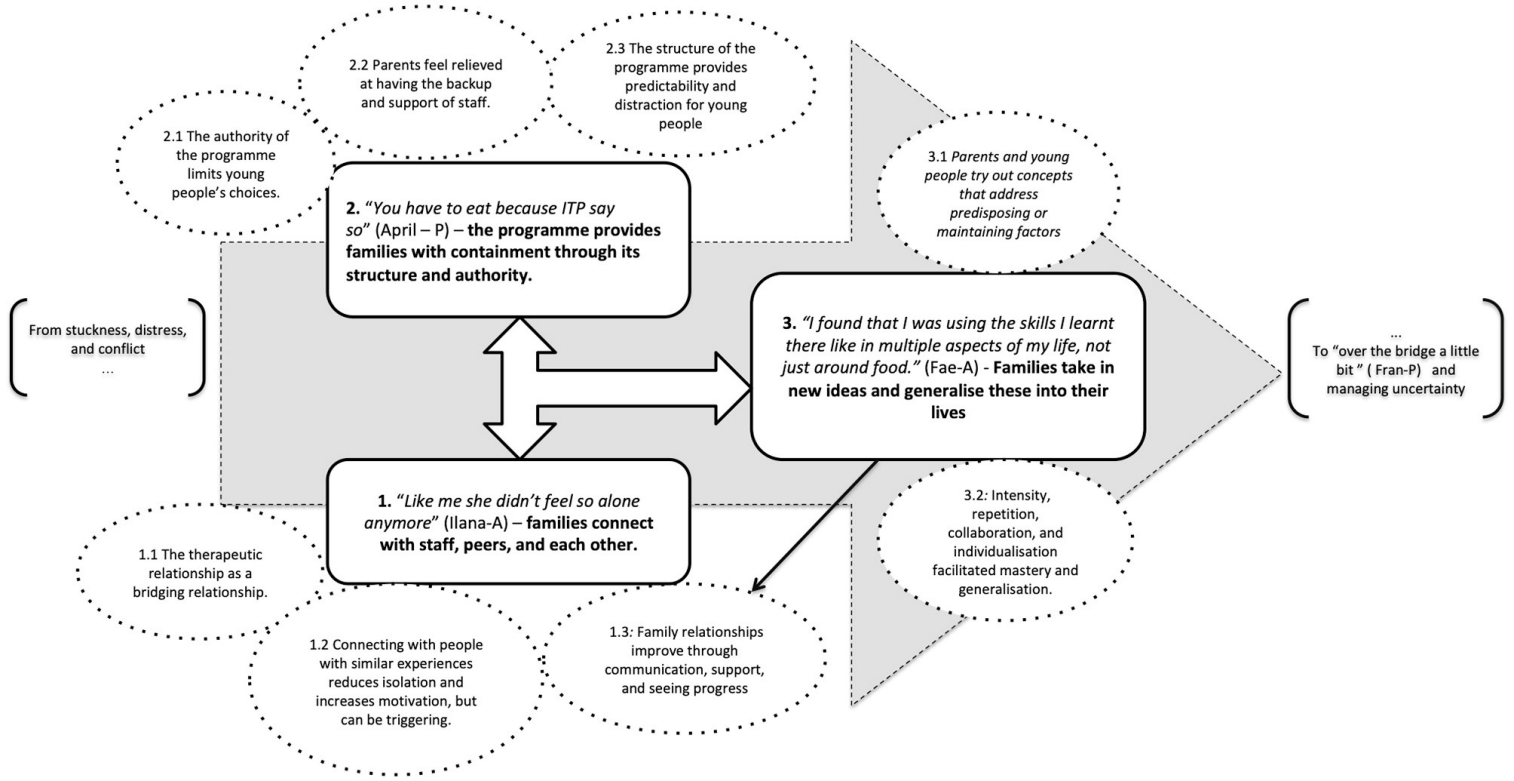
Interconnection of themes creating change.

The three themes are understood as inter-connected, potentiating one another, and all being necessary for therapeutic change (physical, behavioral, psychological, and relational) that lasts beyond the families' involvement with the programme. As described throughout the findings, elements that contributed to positive change also had the potential to have a negative impact for various reasons including processes being too extreme, missing a key element, or becoming counter-productive over time (Figure 1).

Generally, adolescents focused in more detail on the experience of being in the day programme itself. Parents generally took a broader view, talking more about the past, the whole family, the future, their own experiences as parents, and their views on broader UK National Health Service (NHS) treatment provision. Overall, there was considerable agreement between individual adolescents and their parents when they spoke about the same subject. Where adolescents and their parents did not give similar accounts, these were sometimes contradictory but more often complementary (i.e., they said things that could both be “true” without the other one being “false”); examples of these differences are highlighted below. The authors' sense from looking at the paired interviews was that some families had either spoken about the topics discussed or were able to mentalize effectively about their family member's experience. See Table 2 for all themes and subthemes, with illustrative quotes.

**Table 2:** Themes and subthemes.

**Theme**	**Subthemes**	**Illustrative Quotes**
Theme One: “*Like me she didn't feel so alone anymore”* (Ilana-A)—families connect with staff, peers, and each other.	*1.1 “You ended up getting into a relationship with them, which was very helpful.”* (Helen-P)—The therapeutic relationship is a bridging relationship.	“*We've certainly found the people that we've worked uhm, our mini-team, a lot of the people here, uhm, were just, yeah, amazing, we could not have asked for, uhm, anything more.”* (Bridget-P)
	*1.2: “It's good because you kind of feel like in a bit of a community, like a group struggle almost” (Bella-A)*—Connecting with people with similar experiences reduces isolation and increases motivation, but can be triggering.	“* Uhm, starting to just reconnect with other people and as well, noticing like, you're not alone in it and that they're going through the same thing.”* (Fae-A)
		“*Anything- anything sort of where you just feel ‘god', when you listen to other parents, you think ‘well …it's only- it's not just me', you know, everything they were saying would be how we felt as well so that was- it was really reassuring, that was really helpful.”* (Fran-P)
	*1.3 “I had quite a good relationship with my parents. Uhm, but I think we're closer now”* (Gemma-A)—Family relationships improved through communication, support, and seeing progress.	“*And also because the eating disorder was being squashed as it were, or being you know, there was more of her that came back out I think. The old person. Uhm, and so you were able to connect with- with that person again.”* (Helen-P)
Theme two: “*You have to eat because ITP say so*” (April-P)—the programme provides families with containment through its structure and authority.	*2.1 “If there weren't like, strict boundaries, I just, I just wouldn't have done it”* (Daniela-A)—The authority of the programme limits adolescents' choices.	“*Uhm...the fact that it was almost kind of like, I'd describe it as tough love, because obviously they cared and you knew that they cared, so you knew you had people there for you, but at the same time, there were dire consequences AKA like fortisip [nutritional supplement drink] if you didn't do what they said. So knowing that you had no choice made it a lot easier*”. (Ilana-A)
	*2.2 “It was like having a massive weight lifted”* (Dawn-P)—Parents feel relieved at having the backup and support of staff.	“*Well, she did it once, she was like ‘I don't care, I'll just have it [replacement with fortisip]', and then she was like ‘gosh, I drank it all day I'm not going to do that again' [laughter], so yeah”* (Eleanor-P)
	*2.3 “That structure is really... crucial”* (Helen-P)—The structure of the programme provides predictability for adolescents	“*Because before I came to ITP, well I had been in hospital last year, and then for the first half of this year I was just at home with no structure which wasn't very helpful.”* (Alice-A)
Theme three: “*I found that I was using the skills I learnt there like in multiple aspects of my life, not just around food.”* (Fae-A)—Families take in new ideas and generalize these into their lives.	*3.1 “It's maybe not in my nature but I'm having- I'm teaching myself.”* (Fran-P)—Parents and adolescents try out concepts that address predisposing or maintaining factors	“*So it's definitely made me more aware that you know, it can't just be food, it's got to be [pause] proper food. If that makes any sense?”* (Eleanor-P)
	*3.2 “Sort of making little steps”* (Clara-A)–Intensity, repetition, collaboration, and individualization facilitated mastery and generalization.	“*It was definitely helpful, I think, looking back on it now, because had I not eaten my fear foods, had I still been afraid of them, I probably wouldn't have moved forward–I probably would have just stayed where I was, still tried to negotiate with food, but the fact that ITP and my parents challenged me so much…is–it was really helpful. Obviously at the time I didn't think so.”* (Bella-A)

Theme one: “*Like me, she didn't feel so alone anymore*” (Ilana-A)—families connect with staff, peers, and each other.

The narratives of adolescents and parents suggest that isolation was a significant issue before ITP, whether pre-dating or resulting from their eating difficulties. Struggles were named or implied by all participants. For many, the conflict and struggle were initially between the adolescent and their parents (and sometimes staff), but shifted over time for many to a struggle with AN itself. Adolescents with more reported progress also tend to describe more (re-)connections.

*Subtheme 1.1: “You ended up getting into a relationship with them, which was very helpful.” (Helen-P)*—The therapeutic relationship is a bridging relationship.

Narratives about staff were overwhelmingly positive. Participants spoke about the whole staff team as well as their ‘mini-teams' (their individual and family therapists) in a way that suggested a positive therapeutic milieu in the programme and particularly close bonds with their two assigned staff members. Bella (A) reported that “*all the people were really nice, they never said anything negative to you, they were very fair to you…They really listened to you”* and added, “*I think it built up my confidence bit by bit, and on the trust side of things”*. Bella (A) contrasted this with previous experiences “*in CAMHS it was just refeeding, it wasn't about listening to me, and I felt very unheard”*.

The adolescents who were able to speak more openly with parents in family sessions often attributed this to feeling safe about opening up in their therapy as a first step before saying it in family sessions. Ilana (A) explained: “*I knew that they like cared about helping me get better, I guess, so it just- I was able to kind of buildup that trust*”. Some adolescents spoke of their therapeutic relationship as motivating. Clara (A) said: “*I really respected them [staff] and trusted them and stuff so I didn't want to let people down*”. This desire to please staff could have a negative impact as well. For example, Fae (A) said “*I think I struggled a bit at the beginning because I felt like I was letting my team down if I was like, not gaining, or not complying completely so yeah […] I felt like I had to sort of sometimes pretend that I was doing better than I was*”.

Alice (A) had a different experience of the therapeutic relationship; she stated “*Uhm [long pause] I felt my psychologist lost faith in me”* and explained, “*It's just [pause] one day, it was like–it was almost like she thought I was a fake”*. It is unclear where this feeling came from; Alice (A) appeared to blame herself in part saying “*I'm not very good at being vulnerable”*. This lack of therapeutic connection likely reflected and perpetuated Alice's sense of disconnection and mistrust.

Some parents noted the importance of the therapeutic relationship not just for their daughters but also for them. They spoke of feeling respected and on the same team as professionals in their struggle with the illness. Carol (P), who had felt blamed by previous services, noted an immediate difference in the perceived attitude of staff toward her and her husband:

“[I] *Immediately felt just in safer hands […] Because I[…] I respect and trust them and I knew that they didn't think that about me [crying] […] we felt very respected and as though we were part of what was going on and, uhm, that we were all going to be working together to help Clara*.”

Carol (P) described self-blame as “*such a waste of time”* and how letting go of self-blame in ITP freed her up to focus on recovery: “*It made us have uhm room for- to care for Clara better... because you're not being sort of dragged back all the time […] by just feeling rubbish.”*

*Subtheme 1.2: “It's good because you kind of feel like in a bit of a community, like a group struggle almost”* (Bella-A)—Connecting with people with similar experiences reduces isolation and increases motivation, but can be triggering.

Meeting people experiencing similar difficulties was described as largely helpful by parents and adolescents, despite some reservations about this aspect of the programme before starting. Clara (A*) stated, “it's a kind of community, and obviously the community can be really negative but I think it can be positive if you can help each other.”* Ilana (A) was clear that fighting her eating disorder alongside others was a key part of her progress, through helpful accountability among the adolescents, and a bit of social pressure not to let each other down:

*I never really met other people with similar difficulties who were kind of trying to fix them. So that was a motivation. […] like...people- there were people who would hold you to it if you didn't gain weight, you know, you felt like you were kind of pressured in a way, I guess... because they knew what you were trying to do and they knew the situation you were in, so therefore you were held accountable*. (Ilana-A)

Parents also reported benefiting from being around other parents whose children had anorexia nervosa for similar and different reasons to their children. Like them, there was a sense of being less alone in their experiences and learning from each other.

“*Yes, yeah, definitely [pause] because you think you're on your own, don't you? Because the rest of the journey is all by yourself. And then to suddenly to be with people, not necessarily all at the same part, but you've all gone along the same route [pause] and to kind of realize you're not all on our own, it's you know, everyone is going through the same thing.” (Eleanor-P)*

An aspect mentioned more by parents than adolescents is that seeing families with the same difficulties helped them reduce their self-blame as parents, as well as any blame they or their partner felt toward their child. Helen (P) described how her anorexia nervosa was a “*meritocracy*” because it is “*such an illness that it's the same for everyone”*. Fran (P) said that “*seeing everybody's shapes and sizes […] just makes you realize that it isn't anything we've done, it's just how we've ended up and we just need to deal with it. […] that was really helpful.”* Some adolescents like Fae (A) also thought about this experience for their parents: “*I think it was nice for them having like, the parents group. Because they were able to again recognize that other families were in the same position.”*

Several participants indicated that being around others was most helpful when they were earlier in the journey and others were further along. It allowed them to see a roadmap of how the adolescents might also recover, which increased hope and motivation. The flipside of this benefit for those earlier on in their treatment and recovery was that it could be unhelpful for those further along, who may have been finding the presence of acutely unwell adolescents triggering. Greg (P) noted: “*[Gemma (A)] would say ‘well I'm not as thin as them […] so to be here, I need to be thinner. So therefore, why should I eat?”'*. Comparisons and triggering comments could have a negative impact throughout the programme. Ilana (A) explained, “*obviously anorexia is like a really competitive illness and it can be really toxic at some times* “ and that “*some people liked the fact that they were slimmer than other people*”, and Bella (A) summarized this idea: “*I constantly felt like I was kind of looking at my ideal body image”*.

*Subtheme 1.3: “I had quite a good relationship with my parents. Uhm, but I think we're closer now” (Gemma-A)—*Family relationships improve through communication, support, and seeing progress.

Parents tended to highlight that the whole family had suffered and was battling anorexia nervosa together; for example, Helen (P) described that “*an advantage of ITP*” compared to inpatient admission “*is that you're on the journey with them*.” Adolescents were more likely than their parents to conceptualize it as their individual struggle. Most participants noted a significant improvement in their parent–child relationships, which had been negatively impacted by the illness. Carol (P) explained, “*So much of our relationship before was awful because of the eating disorder. So, when that goes then we're getting back to more of our how we were before. [laughter] Which is, which is really good”*. (Carol-P).

This change was generally conceptualized more as an outcome of other changes rather than as a process of change, but some participants hinted at its contribution to change through a decrease in conflict enabling parents to care better for their daughters. Helen (P) noted that “*Well it's- it's- well it's- it makes it much easier to care for somebody, than if being shouted and screamed at and treated negatively”*. Fae (A) highlighted how this might also increase motivation to recover:

“*I think they're a lot more comforting and they don't get angry, whereas before, I guess they would get really frustrated if I wasn't eating a meal, but now it's a bit more like they'll listen to me first and try to understand rather than getting annoyed. […] I think it's made me closer to them, so I kind of feel a bit more motivation to do it in order to help them.”* (Fae-A)

Participants noted that they had to spend more time together in the process of treatment. Fae (A) and Fran (P) spoke similarly about how spending more time together brought them closer, with Fran wondering how to maintain emotional closeness without food:

“*I think we've got closer because… naturally I've spent more time with them … around meals and after meals, so yeah. And also, we go for like walks and stuff and just get us to talk about things we wouldn't talk about before*.” (Fae-A)

“*I'm wondering how we're going to … have the relationship but just not have the food involved in it somehow and we need to be able to still be this close as we now are*.” (Fran-P)

April (P) and Alice (A) had differing views on the intensity of their time together. Alice (A) stated that “*if anything, we're closer […] just because we've had to spend like a lot of intense time together”* whereas April (P) expressed that this increased time was detrimental to their relationship: “*because that's not normal, you know, normally they go to school, they come home, you're not 24/7 trying to get somebody to […] eat something they don't want”*. Additionally, April (P) noted that “*sometimes when some of those boundaries have come down, it's hard to put them back up. So you've said things, both of you, […] you've shown emotion that you never probably exposed to each other before”*.

Theme two: “*You have to eat because ITP say so*” (April – P)—the programme provides families with containment through its structure and authority.

The families who attend ITP have experienced treatment non-response and all typically presented with high levels of anxiety. Some parents described the process of their daughter's deterioration as traumatic. As a result, families needed a sense of containment, to feel that there was a clear structure around them, with established goals, supported by people with expertise in adolescent anorexia nervosa.

*Subtheme 2.1: “If there weren't like, strict boundaries, I just, I just wouldn't have done it”* (Daniela-A)—The authority of the programme limits adolescents' choices.

Adolescents and parents shared some interesting insights into how adolescents were enabled, or not enabled, to accept their meal plan, both within the programme and at home. Typically, adolescents were quicker to eat meals and snacks provided by the professionals within the service context than the food provided by parents at home. Adolescents either expressed a belief that they had no choice but to eat, or that they had limited choices: the choice to eat their meal plan, have it replaced with a nutritional supplement drink, or be suspended from the programme and potentially face admission to an inpatient unit. Gemma (A) highlights that there is a choice, but that one is quite obviously better from her perspective:

“*Uhm, I mean [pause] yeah...it's, yeah [laugh]... I think it's good that they have it [replacement with fortisip] there because otherwise there would- like, people would just not eat, and they wouldn't get better, so I think like if you're sort of, not forced to eat, but like, if you have either one choice, or a choice that's worse, then you're going to choose the better one, even if you don't wanna, so I think it like helps people, even if they don't realize”*. (Gemma-A)

Daniela (A) and her mother had similar narratives around the idea of not having a choice. Dawn (P) expands that it took the pressure off their relationship:

“*So- and I think that that is- that's the magic, really, uhm, is that you take away the responsibility of the child to make the decision. And I think that that's a massive relief for her actually [pause] yeah, and it also- yeah, took the pressure off of our relationship, me kind of going ‘come on, you've got to eat, you've got to eat, you've got to eat' because Daniela [pause] no longer had any- as far as she could see she had no choice.”* (Dawn-P).

Bella described an incident early in ITP where she did not eat a meal, and eventually had a replacement after two members of staff encouraged her to, which she described as building trust in staff:

“*I didn't regret coming, but I was definitely in shock … the determination that they wouldn't give up. [...] Uhm, I think it just–I think it taught me to [pause] trust others a little bit more. [...] In a way that you, that you don't have any choice, so you might as well get it over with.”* (Bella-A)

Clara (A) described a very different response to the rules of ITP and maintained that no one can physically make another human being eat. “*I...that's a tricky one for me to answer, I think... because I [felt like I] was force-fed for a while. I dunno about- I dunno about that. I don't think it [the consequence of meal replacement with fortisip] works, but I can't see how you get someone to eat, so...I dunno.”* (Clara-A)

The parents and adolescents also alluded to the possibility that the strict rules and clear consequences allowed the part of the adolescent that wanted to recover to argue with or appease the part of them that did not. This promoted separation from, or increased ability to manage, their eating disorder cognitions and behaviors. Many spoke of finding eating easier once they felt they had no choice, which appeared to pave the way for other processes described across the themes. Ilana (A) explained how this sense of having no choice helped her: “*you weren't giving yourself such a like mental battle every time you had to eat.”*

“*But in that moment, it was- it seemed to us-me was she had wanted that all along... maybe it was the fact that it was- she could say “well, I...it's ITP that are telling me to this”... and so it's- and it was maybe a way of that she could argue with the anorexia.” (G*reg-P)

Eleanor (P) hypothesized that not having a choice reduced her daughter's guilt: “*Whereas here, the food she had to eat, there was no choice, you had to eat it. And because she didn't have a choice, she couldn't feel that guilty about it. Because it wasn't her that chose to eat it*.” Her daughter Evie (A) framed it slightly differently, saying “*Uhm, it in a way made me want to finish, because I didn't want the added calories of the fortisip which is more than the meal itself* ”, but does not refer to it being “easier” or less guilt-inducing. Daniela (A) specifically said that not having a choice did not reduce her guilt, but did allow her to argue: “*well every time I ate I felt guilty, so. I don't think it helped around that, but the fact that you don't have a choice means you can just say ‘well, there's nothing I can do about it, so...'*”

*Subtheme 2.2: “It was like having a massive weight lifted”* (Dawn-P)—Parents feel relieved at having the backup and support of staff.

Parents expressed a strong sense of relief at their child being in “*safe hands*” (April, Fran, and Carol -P) and knowing that they will eat while at the programme. Parents were initially reassured by the sense of having a “*back-up*” (Helen-P) in terms of knowing that staff will offer support, which seemed to lend parents authority: “*I think it just gave us authority to be able to say this is what needs to be done*.” (Greg-P).

Fran (P) described realizing that staff could replace missed meals at home with fortisip: “*And then I just knew that...well, my savior is that I can inform them [ITP] … that really was very helpful”*. Evie (A) reported some ambivalence about having food missed at home replaced in the programme, saying “*Uhm, it was OK because then they were making you like, replace the meal you missed, but then the bad thing was that you were replacing the meal that you intentionally missed [laughter]”*. This ambivalence likely reflected a wider ambivalence about recovery. This strategy generally seemed to lead to far fewer arguments about food at home, which was a relief for both and seemed to take some pressure off the parent–child relationship.

Some parents also valued having some time to themselves after long periods of intense supervision at home before the programme, particularly not having to do all the meals with their daughter. Eleanor (P) reported, “*it was good... uhm, because at least I knew that the meals she had here were meals that I didn't have to fight with her [laughter], that somebody else was doing that for a bit*”.

Some parents expressed some guilt at valuing this break but also considered the benefits in terms of other family relationships, as well as how their wellbeing impacts their children.

“*I think as parents it gave us, uhm, in the early days just some respite, uhm, it was very intense and very...very difficult at home.”* (Bridget-P)

“*Uhm, yeah, and sort of helped me to give me a little bit of free time as well. Which I kind of enjoyed [...] So it, it worked well for me as well. I felt guilty–I felt guilty about it but it did give me some free time.”* (Fran-P)

However, parents could also feel disempowered in relation to the authority of the programme and its staff. A couple of parents expressed frustration or dismay that staff seemed to have a “*magic*” ability to get adolescents to eat and felt that they as parents who know their daughters intimately should be more able to do so.

“*Well, that made me feel a bit rubbish because I thought ‘what is it they can do that I can't do?' you know? […] because at first I thought to myself, ‘well, they are the experts, and that's probably why they can make her do these things and that's maybe why I can't', but at the same time, I thought ‘no, because I'm still- we're still her parents, we should be able to do even better than them because we know her so well', you know.”* (Fran-P)

There was a sense that ITP staff's authority had only been lent out to them, and they were not sure they had their own equivalent authority with regards to their daughter's eating disorder.

“*I start negotiating, [laughter] I know I shouldn't, but then I'm like, because she says I'm not eating, then I'm like ‘I've got to get you to have something' so what's the least fearful thing I can get you to have so you're still keeping your calories up, which then isn't tackling anything else, but it- [sigh] so yeah, I dunno.”* (Eleanor-P)

The anticipation of no longer having the backup of the programme after discharge was also a source of concern, particularly for Alice (A) and April (P) who had not seen as much change during the programme as they had hoped: “*I think we're all a bit–even Alice is a bit nervous about when that threat isn't there.” (April-P)*.

*Subtheme 2.3: “That structure is really... crucial”(Helen-P*)—The structure of the programme provides predictability and distraction for adolescents

Most adolescents stated that having a structure to their days with predictable mealtimes and expectations about eating was beneficial. Most parents also noted the significance of the structure itself in benefitting adolescents, particularly in keeping them busy and knowing what would happen when.

“*So I suddenly– I realized that that's what ITP did. It provided the structure, even though she didn't want it, she was reassured by the fact that somebody was going to stick to the rules regardless. [...] I suppose it's the structure, the structure of the whole thing...so- and the immovable kind of boundaries*.” (Dawn-P)

Some adolescents noted that they had insufficient structure before coming to the programme, which meant their lives were increasingly focused on food. The regular mealtimes in the programme meant that adolescents knew when food would arrive. Interestingly, combined with the distraction of groups and education, this seemed to mean that their lives felt less focused on food, despite attending a day programme for restrictive eating disorders.

“*It meant that I had things to do. Like, I had to do them, I didn't have the option to... not do them [AC: uh-hmm] and I knew... and I know when a meal is coming and like often, what it's going to be. [...] Uhm, it just makes me feel like a bit more like, secure, and like, in the know*.” (Alice-A)

Adolescents were clear that at times when there was less structure such as less structured therapeutic group sessions, it “*let your [pause] your mind wander*” (Bella-A). It was implied that the mind would turn to thoughts relating to food or weight, which in turn could temporarily increase distress.

Theme three: “*I found that I was using the skills I learnt there like in multiple aspects of my life, not just around food.”* (Fae-A)—Families take in new ideas and generalize these into their lives.

Adolescents and parents talked about how changes made within the programme were often a first step toward making them back in the “real world”. As Fran (P) explained, “*we always use it and go back and say well you did it in ITP, so we can do it, you know*”. This process was often promoted by the intensity of the programme. Several specific elements, such as certain groups and eating meals out with staff, were mentioned as particularly useful in this process and can be understood as addressing predisposing and maintaining factors of relevance to their eating disorder. Participants also described the usefulness of ITP being time-limited, as well as the combination of individualized care, that was collaboratively agreed upon with their “mini-team”, provided within the common structure with the same expectations for everyone. This was described as all helping participants be able to get “back to life” more quickly.

*Subtheme 3.1 “It's maybe not in my nature but I'm having- I'm teaching myself.” (Fran-P)*—Parents and adolescents try out concepts that address predisposing or maintaining factors.

The idea of ITP being a place to make a small change that could then be generalized to the “real world” was articulated by adolescents and parents alike. Participants often described these changes in the “real world” with excitement and pride:

*Gemma came in and was so chuffed to have been, and sort of, “I've been out with my friends for the first time, yeah, somewhere to eat, uhm, I eat- and I got, I had a coke and a sprite because why wouldn't you, it's bottomless drinks” [laughter]. And I was sort of gobsmacked because I can't even remember her ever drinking a coke and sprite...before, rather than-never mind post-anorexia- well I say post, we're still in recovery, but [pause] yeah, so lots of positives.”* (Greg-P)

Adolescents spoke about applying skills from the groups to their lives, particularly those related to perfectionism and relationships and social skills. Fae (A) explained, “*I found perfectionism group really helpful, because perfectionism is something I've struggled with like, my whole life. [...] It was nice kind of challenging that head on*.”

“*I was like very determined to not [engage in groups], to be honest, and then you have to do, like homework and that kind of thing, although I didn't usually do it, I- I did think about it a lot, and ended up accidentally applying it to life so [...] I'd be going into a social situation and I'd have my headphones on, and I'd be like ‘No! that's a shield! [laughter] You need to interact with people'.”* (Daniela-A)

“*Uhm, all the different uhm, like, not classes, but the different like, RO DBT and perfectionism and like, all those kinda things [groups], I felt were helpful in like helping me understand my ways of thinking, ways to cope with it. [...] I'm trying to be less unhealthy perfectionist.”* (Evie-A)

Most adolescents had been out of school or even if in school, had felt disconnected from friends. Several adolescents and parents reported that connecting with other adolescents on the programme got them back into the habit of socializing and built their confidence in this respect, allowing them to reconnect with their friends outside the programme.

There was also a sense from some that the programme helped them to reengage in the world: As Dawn (P) described:

“*She got into the sea [...] we were just bobbing around in the waves, and she just went she just said ‘Mummy, I just feel like I'm back' and so, you know, it was a really nice moment where I thought yeah she said ‘I haven't been in the'- I think she was like, ‘I'm back in the sea', but...actually, I kind of felt like ‘oh my god, but you are back' because she hadn't been in the sea for a whole year, [...] it was a nice moment*.” (Dawn-P)

Participants did not mention generalizing skills or ideas from individual therapy; this does not necessarily mean that it did not occur; perhaps relational aspects of individual therapy eclipsed other aspects.

*Subtheme 3.2 “Sort of making little steps” (Clara-A)*—Intensity, repetition, collaboration, and individualization facilitated mastery and generalization.

Participants reported that the intensity of the programme was needed for the benefits described above to be seen; Carol (P) argued “*the more intense the better [laughter] as far as I was concerned. I mean I think it's the most intense illness so, and it's just, you need all the help you can get*.” A couple of participants wondered about having more intensity at the start because parents are usually so tired, or a more gradual build-up to full-time at the start for adolescents not to be overwhelmed.

Several participants wished that attendance could be for longer than the typical “*two rounds”* (one UK school term), but others seemed to think that this gave a focus and a sense of needing to make the most of opportunities while on the programme. Some also highlighted the benefit of being able to get back into life fairly quickly.

“*I think [ITP being brief] is important because obviously they can't keep everyone there forever and it made it better, because when I did have to go, I felt like OK, I got a lot out of it, maybe not as much as I could have done, but...you know, if it had been less intense I wouldn't have got as much out of it. And it also means you can get back to normal life a lot quicker.”* (Fae-A)

Some participants gradually reduced their attendance intensity and returned part-time to school; those for whom this was not possible felt this would have been beneficial.

“*I think for us, actually having that very intensive period, having it very much centered about being here, and just, and just really sort of pushing it all together in this very condensed period was a really good thing*.” (Bridget-P)

“*I think it would have been good if I'd, like, a little bit more of a step-down whereas it was a bit like ‘you're here and then you're not here ever' but...yeah...there wasn't anything that could be done in my situation, and I know that everyone else did have that.”* (Daniela-A)

While adolescents were clear that their difficulties were not just about food, the reintroduction of feared food items was viewed as important. Fae (A) explained that she needed the day programme “*because I think the things that I'd avoided for so long that I felt like I couldn't find the motivation to try it, if I was just alone at home. I needed to be in a like, supported environment*”.

Most adolescents spoke of finding gradual steps and repetition of food-related challenges useful in mastering these challenges. Clara (A) described it as “*sort of making little steps. And you know, sometimes it would go that way sometimes it would go that way, but [pause] and that that was actually a way for me to build my own strength around things”* and that “*It just seemed to work...just kind of slow, yeah*.” Fae (A) felt that more repetition would have benefited her: “*I think when we went out for meals, that was really helpful, and it would have been more helpful to maybe do that more often. Or like, repeat certain challenges, because doing them once usually doesn't conquer the fear*.”

Often, weight gain and food-related achievements were valued for the other changes they enabled, such as increased independence; Bella (A) explained that “*I feel like at the start it had to be no choice and then that slowly meant that like I could like have more choices about what I did”*. Some adolescents were particularly positive about having meals out with staff because of their goals.

“*I went out to eat quite a lot, because that was one of my targets to really, really feel comfortable. Not just going out with a group to eat, but to really feel comfortable around eating in cafes and all that.”* (Bella-A)

Some adolescents with many fear foods found the early weeks of ITP challenging and even overwhelming. Evie (A) said “*I don't know if it's just me, but all the food list was like, foods I'm like, scared of [pause] so that was quite hard”* and suggested that “*I think, like, maybe a- one fear food a day, would have like, helped you build up a bit more than stressed you out*”. Conversely, Gemma (A) reported that “*I came to ITP, literally everything was my fear food, but then because they gave it, because they gave it to us at lunch, then I just got over*.”

## Discussion

This study aimed to explore adolescent and parent experiences of day programme treatment and how change is perceived to occur. Parent and adolescent narratives tended to be complimentary, rather than contradictory; however, they had different foci at times. The analysis suggested several interrelated processes that impacted change and progress during day programme treatment. Three interconnected themes were interpreted from the data: (1) families connecting with staff, peers, and each other; (2) the programme providing containment through structure and authority; and (3) families taking in new ideas and generalizing them out to the “real world”.

Helpful aspects of the programme were frequently related to what families felt they had lacked before it, as well as difficult historical experiences related to treatment non-response. Notable important elements included the intensity of the programme; the containment created by staff authority; the provision of clear information about anorexia nervosa; practical support for parents to address gaps in knowledge, and individual support for the adolescents to enable better use of family sessions. This helped in multifaceted ways including establishing connections between adolescents and staff, other adolescents with anorexia nervosa (and for parents), and family members; establishing rules and structures to facilitate adolescents to eat and gain weight; gradual and repeated food challenges; and group therapies focusing on social skills, perfectionism, and anxiety. Together, these processes encouraged a change in the adolescent's relationship with their eating disorder and their family members, which promoted more negative views of AN and more distance and ability to manage it.

Some unhelpful processes were also identified. Most typically resulted from otherwise helpful processes. Often, they were either reflecting “flipsides' of these processes, or the application of helpful strategies without the crucial relational element. For example, staff firmness, which participants often found helpful, could be experienced as harshness when adolescents did not feel they were being related to as individuals. Staff authority and support could inadvertently create dependence among families, which made the programme ending very challenging; the presence of adolescents was positive overall but could not be made entirely free of comparisons and competition.

The way connection (and re-connection) helped was conceptualized slightly differently by the adolescents compared to the parents. Parents described increased connection helping the difficulties feel more shared between parent and adolescent. Slightly differently, the narratives of the adolescents were that connection helped them move away from isolation to a more supported, but still individual, struggle with the illness. The extent to which this was present varied between narratives, where adolescents with more self-reported progress also tended to describe more (re-)connection.

While no other qualitative data about adolescent day programme experience are available to directly compare the current findings too, several similarities are noted between the current findings and recent qualitative studies of adolescent and parent experiences of higher intensity treatment for adolescent anorexia nervosa (Nilsen et al., 2021; Baudinet et al., 2023). Nilsen and colleagues (Nilsen et al., 2021) found that adolescents with anorexia nervosa who experienced inpatient treatment described relief about getting more intense help, a change in reported ambivalence over time, both benefits and challenges related to being with others struggling with similar difficulties, valued staff expertise, observed improvements and challenges in family relationships, and fear of discharge back to services that were not felt to have helped previously. Similarly, in intensive outpatient multi-family therapy, intensity, connection, comparisons, and skill building were all perceived as change mechanisms that promoted hope, mentalization, self-efficacy, and change (Baudinet et al., 2021a, 2023).

The importance of reducing isolation and increasing safety and containment links with the idea of epistemic trust (how much one trusts and is willing to consider information provided by others Fonagy and Allison, 2014) and “relational containment” (Wallis et al., 2017), both of which have been described as important processes of change in outpatient family treatment (Jewell et al., 2016). This also fits with the emphasis placed on engagement and formulation in FT-AN (Eisler et al., 2016; Baudinet et al., 2021b, 2022). Where the current findings extend this data, is by emphasizing the increased role that a day programme multi-disciplinary team can have in establishing containment in the more intensive setting. Additionally, the group-based approach concurrently appears to contribute toward reducing isolation. A recent meta-synthesis reported that carers of people with eating disorders can experience guilt and self-blame; shock, anger, helplessness, and mistrust; and worry and rumination (Fox et al., 2017). The day programme setting and staff approach appear to offer predictability and kindness, essentially creating a “safe base” for treatment. The current data suggest that the structure and staff approach contains both adolescents and parents, which, in turn, supports increased containment between parents and adolescents.

At each step in this process, containment seems to lead to relational safety, which may in turn be associated with an increase in mentalization in adolescents and parents, enabling them to think about their and each other's inner worlds. Impaired mentalizing and, more recently, epistemic mistrust have been theorized to link to eating disorder presentations, positing that they are central processes in the development and maintenance of anorexia nervosa (Robinson et al., 2019). These conjectures are offered tentatively and further quantitative and qualitative research is needed to better understand these potential mechanisms and how day programme, and other intensive treatments, may address this.

### Strengths and limitations

A strength of this study is that it begins to address the relative dearth of qualitative research into the views of families attending day programmes for adolescent restrictive eating disorders. The use of dyadic interview analysis of parent–adolescent paired interviews allowed for an in-depth systemic understanding of their experiences during the programme. This rich data will be of interest to the growing number of day programme providers in the UK and abroad.

The dyadic approach, while providing unique insights, means that the analysis is based on data from only 9 families. Additionally, this small sample was recruited from a single service and the reader will need to judge the degree to which the findings are relevant to their own service context. Researchers did not gather information about participant ethnicity, which would have been interesting given the growing literature addressing ethnic and cultural differences in eating disorder presentations.

### Clinical implications and future directions

Participants were overwhelmingly positive about their interactions with other people experiencing similar difficulties, despite many also having been concerned about this aspect before the programme. Similar but more limited benefits of group work with peers in multi-family therapy have been described elsewhere (Voriadaki et al., 2015; Baudinet et al., 2023), pointing to the need to explore the benefits of peer support more thoroughly for adolescents with anorexia nervosa. Participants particularly described the benefit of being around adolescents or parents further ahead in the process of recovery. However, several participants reflected that for those further in recovery, it was triggering to see those earlier on in the process. Rhodes and colleagues (Rhodes et al., 2009) reported several benefits of adding “parent-to-parent consultations” to standard family-based treatment. These consultations consisted of a single session between a parent who had completed treatment and a parent beginning treatment. The finding that adolescents value an equivalent experience only further enhances the need to explore the use and impact of peer support more thoroughly and rigorously.

The current findings highlight that a very firm approach may be needed to promote change for adolescents and parents who need greater treatment intensity than can be provided in first-line outpatient treatment. However, this approach can also be unhelpful if a foundation of trust and collaboration is not initially established. The issue of control, responsibility, and collaboration within treatments for anorexia nervosa also needs to be further examined. Further research is needed to establish what other factors may contribute to effective collaboration. Furthermore, it would be useful to explore what other factors that may be contributing toward the establishment of effective collaboration for this group specifically. Alliance with both adolescents and parents has been shown to predict improved outcomes in FT-AN (Jewell et al., 2021). A quantitative exploration of this in the day programme setting is also indicated from the current data.

## Conclusion

Intensive outpatient treatment for anorexia nervosa was acceptable to adolescents and their parents, who noted many core interrelated processes that enabled them to make meaningful changes. While the behavioral aspects of the treatment were deemed essential, these could not lead to change beyond the programme without strong therapeutic relationships.

## Data availability statement

Ethics approval was for the data to only be used in the current study. Further inquries about the datasets should be directed to amy.colla@nelft.nhs.uk.

## Ethics statement

The studies involving human participants were reviewed and approved by London-Hampstead Ethics Committee. Written informed consent to participate in this study was provided by the patient/participants' OR patient/participants legal guardian/next of kin.

## Author contributions

Recruitment conducted by JB. Data collection and analysis conducted by AC. The manuscript was drafted by AC and JB and collaborating authors HS, PC, and EG read and approved the final manuscript. All authors contributed to the article and approved the submitted version.

## References

[B1] American Psychiatric Association (2013). Diagnostic and Statistical Manual of Mental Disorders: DSM-5. Washington, D.C: American Psychiatric Association, 947.

[B2] Baudinet J. Eisler I. Dawson L. Simic M. Schmidt U. (2021a). Multi-family therapy for eating disorders: a systematic scoping review of the quantitative and qualitative findings. Int. J. Eat. Disord. 54, 2095–2120. 10.1002/eat.23616 34672007 PMC9298280

[B3] Baudinet J. Eisler I. Konstantellou A. Hunt T. Kassamali F. McLaughlin N.. (2023). Perceived change mechanisms in multi-family therapy for anorexia nervosa: a qualitative follow-up study of adolescent and parent experiences. Eur. Eating Disord. Rev. 10.1002/erv.3006. [Epub ahead of print]. 37415392

[B4] Baudinet J. Simic M. (2021). Adolescent eating disorder day programme treatment models and outcomes: a systematic scoping review. Front. Psychiatry. 12, 539. 10.3389/fpsyt.2021.652604 33995149 PMC8116630

[B5] Baudinet J. Simic M. Eisler I. (2021b). Formulation in eating disorder focused family therapy: why, when and how? J. Eat Disord. 9, 97. 10.1186/s40337-021-00451-3 34376258 PMC8353776

[B6] Baudinet J. Simic M. Eisler I. (2022). “From treatment models to manuals: maudsley single- and multi-family therapy for adolescent eating disorders,” in Systemic Approaches to Manuals. 1st edition, Mariotti M. Saba G. Stratton P. (eds.). Cham: Springer, 349–72.

[B7] Baudinet J. Simic M. Griffiths H. Donnelly C. Stewart C. Goddard E.. (2020). Targeting maladaptive overcontrol with radically open dialectical behaviour therapy in a day programme for adolescents with restrictive eating disorders: an uncontrolled case series. J Eat Disord. 8, 68. 10.1186/s40337-020-00338-9 33292696 PMC7663904

[B8] Braun V. Clarke V. (2006). Using thematic analysis in psychology. Qual. Res. Psychol. 3, 77–101. 10.1191/1478088706qp063oa

[B9] Braun V. Clarke V. (2012). “Thematic analysis,” in APA handbook of Research Methods in Psychology, Vol 2: Research Designs: Quantitative, Qualitative, Neuropsychological, and Biological. Washington, DC, US: American Psychological Association, 57–71.

[B10] Braun V. Clarke V. (2020). One size fits all? What counts as quality practice in (reflexive) thematic analysis? Qual. Res. Psychol. 12, 1–25. 10.1080/14780887.2020.1769238

[B11] Eisikovits Z. Koren C. (2010). Approaches to and outcomes of dyadic interview analysis. Qual. Health Res. 20, 1642–1655. 10.1177/1049732310376520 20663940

[B12] Eisler I. Simic M. Blessitt E. Dodge L. (2016). MCCAED Team. Maudsley Service Manual for Child and Adolescent Eating Disorder. Available online at: https://mccaed.slam.nhs.uk/wp-content/uploads/2019/11/Maudsley-Service-Manual-for-Child-and-Adolescent-Eating-Disorders-July-2016.pdf

[B13] Fischer C. T. (2009). Bracketing in qualitative research: conceptual and practical matters. Psychother. Res. 19, 583–590. 10.1080/10503300902798375 20183407

[B14] Fonagy P. Allison E. (2014). The role of mentalizing and epistemic trust in the therapeutic relationship. Psychotherapy 51, 372–380. 10.1037/a0036505 24773092

[B15] Fox J. R. Dean M. Whittlesea A. (2017). The experience of caring for or living with an individual with an eating disorder: a meta-synthesis of qualitative studies: experience of caring for someone with AN. Clin. Psychol. Psychother. 24, 103–125. 10.1002/cpp.1984 26472481

[B16] Herpertz-Dahlmann B. Schwarte R. Krei M. Egberts K. Warnke A. Wewetzer C.. (2014). Day-patient treatment after short inpatient care versus continued inpatient treatment in adolescents with anorexia nervosa (ANDI): a multicentre, randomised, open-label, non-inferiority trial. Lancet. 383, 1222–1229. 10.1016/S0140-6736(13)62411-3 24439238

[B17] Jacobson D. Mustafa N. (2019). Social identity map: a reflexivity tool for practicing explicit positionality in critical qualitative research. Int. J. Qual. Methods. 1, 18. 10.1177/1609406919870075

[B18] Jewell T. Blessitt E. Stewart C. Simic M. Eisler I. (2016). Family therapy for child and adolescent eating disorders: a critical review. Fam. Process. 55, 577–594. 10.1111/famp.12242 27543373

[B19] Jewell T. Herle M. Serpell L. Eivors A. Simic M. Fonagy P.. (2021). Attachment and mentalization as predictors of outcome in family therapy for adolescent anorexia nervosa. Eur. Child Adolescent Psychiat. 32, 1241–1251. 10.31219/osf.io/ru8sd 34967934 PMC10276078

[B20] Lynch T. R. (2018). Radically Open Dialectical Behavior Therapy: Theory and Practice for Treating Disorders of Overcontrol. Oakland, CA: New Harbinger Publications, 406.

[B21] Maiden Z. Baker L. Espie J. Simic M. Tchanturia K. (2014). Group Cognitive Remediation Therapy for Adolescents withAnorexia Nervosa -The Flexible Thinking Group. Available online at: https://mccaed.slam.nhs.uk/professionals/resources/books-and-manuals/

[B22] Morgan H. G. Hayward A. E. (1988). Clinical assessment of anorexia nervosa. The Morgan-Russell outcome assessment schedule. Br. J. Psychiatry. 152, 367–371. 10.1192/bjp.152.3.367 3167372

[B23] Nilsen J. V. Rø Ø. Halvorsen I. Oddli H. W. Hage T. W. (2021). Family members' reflections upon a family-based inpatient treatment program for adolescent anorexia nervosa: a thematic analysis. J. Eat Disord. 9, 7. 10.1186/s40337-020-00360-x 33407914 PMC7788959

[B24] Parks E. Anderson L. K. Cusack A. (2016). “Adolescent impressions of family involvement in the treatment of eating disorders,” in Innovations in Family Therapy for Eating Disorders: Novel Treatment Developments, Patient Insights, and the Role of Carers, Murray S. B. Anderson L. K. Cohn L. (eds.). New York: Routledge, 208–19.

[B25] Rhodes P. Brown J. Madden S. (2009). The Maudsley model of family-based treatment for anorexia nervosa: a qualitative evaluation of parent-to-parent consultation. J. Marital Fam. Ther. 35, 181–192. 10.1111/j.1752-0606.2009.00115.x 19302516

[B26] Robinson P. Skårderud F. Sommerfeldt B. (2019). “Eating disorders and mentalizing,” in Hunger: Mentalization-based Treatments for Eating Disorders. Cham: Springer International Publishing, 35–49.

[B27] Simic M. Stewart C. S. Eisler I. Baudinet J. Hunt K. O'Brien J.. (2018). Intensive treatment program (ITP): a case series service evaluation of the effectiveness of day patient treatment for adolescents with a restrictive eating disorder. Int. J. Eating Disord. 51, 1261–1269. 10.1002/eat.22959 30265750

[B28] Simic M. Stewart C. S. Konstantellou A. Eisler I. Baudinet J. (2022). From efficacy to effectiveness: child and adolescent eating disorder treatments in the real world (part 1)—treatment course and outcomes. J. Eating Disord. 10, 27. 10.1186/s40337-022-00553-6 35189967 PMC8862310

[B29] Stewart C. S. Baudinet J. Munuve A. Bell A. Konstantellou A. Eisler I.. (2022). From efficacy to effectiveness: child and adolescent eating disorder treatments in the real world (Part 2):7-year follow-up. J. Eat Disord. 10, 14. 10.1186/s40337-022-00535-8 35123587 PMC8817149

[B30] Voriadaki T. Simic M. Espie J. Eisler I. (2015). Intensive multi-family therapy for adolescent anorexia nervosa: adolescents' and parents' day-to-day experiences. J. Fam. Ther. 37, 5–23. 10.1111/1467-6427.12067

[B31] Wallis A. Rhodes P. Dawson L. Miskovic-Wheatley J. Madden S. Touyz S.. (2017). Relational containment: exploring the effect of family-based treatment for anorexia on familial relationships. J Eat Disord. 5, 27. 10.1186/s40337-017-0156-0 28770090 PMC5532769

