# Supplementary material for: Change processes during intensive day programme treatment for adolescent anorexia nervosa: a dyadic interview analysis of adolescent and parent views
Source: Front Psychol. 2023 Aug 10;14:1226605. doi: 10.3389/fpsyg.2023.1226605 (PMC10450334; doi:10.3389/fpsyg.2023.1226605)
Supplement: Supplementary file 1 [file Table_1.docx]

Interview Schedule

## Project title: Change processes in day treatment for adolescent anorexia

# Young Person

## Introductory open questions

**General experience of treatment** (e.g. length/intensity, any suspensions, “turning points”, did they do multi-family Therapy? What was it *like?*…) first impressions

Description of **changes** they have experienced during the programme. [“what’s different for you now compared to before the programme?”]. To what to they **attribute** these changes [“what led to these changes, both within and outside the programme?”]

**Helpful and unhelpful aspects** (e.g. “what was most (un)helpful within and outside of programme? Anything difficult but OK (worth it)?)

**Missing aspects** (“what could the programme have included or done differently that would have helped you/your parent make changes?”)

## Areas to prompt if do not occur spontaneously.

**Intensity** of programme

Part of **group** of other young people with EDs – shared experience, triggers, reduced isolation?

Relationship with parents/**attachment** – how changed as result of ED, and changes during treatment. Includes attachment to professionals.

**Understanding** of anorexia – whether changed, impact on things such as self-blame and guilt.

**Parents** – what YP noticed change in terms of actions and understanding.

**Boundaries, rules, and consequences** – how YP experienced them, if/how Parents

**Specific programme elements**- e.g. groups, famly meal, individual thereapy, family therapy, meal challenges.

New theme(s) emerging from interviews 1-4.

-motivation/hope

# Parent

## Introductory open questions

**General experience of treatment** (e.g. was it straightforward ton get a palce? length/intensity, any suspensions, “turning points”, did they do multi-family Therapy? What was it *like?*…)

Description of **changes** they have experienced or witnessed in their child during the programme. [“what’s different for you now compared to before the programme?”]. To what to they **attribute** these changes[“what led to these changes, both within and outside the programme?”]

**Helpful and unhelpful aspects** – asked through open questions (e.g. “what was helpful? What aspects of life outside the programme impacted your progress?”)

**Missing aspects** (“what could the programme have included or done differently that would have helped you and your child make changes?”)

## Areas to prompt if do not occur spontaneously.

**Intensity** of programme

Part of **group** of other parents of young people with EDs – shared experience, “tips”, reduced isolation? Had they worried about this aspect?

Relationship with young person/**attachment** – how changed as result of ED, and changes during treatment. Includes attachment to professionals.

**Understanding** of anorexia – whether changed, impact on how they experience their child and how they act towards them.

Hypotheses about **what it was like for** **their child**.

**Boundaries, rules, and consequences** – how parents found these, ability to adhere to them, how thy think their child experienced them.

**Actions** – what they have done differently as a result of being in ITP, and what helped them to do this.

New theme(s) emerging from interviews 1-4.

-hope

blameˇ
